# Developing a new small-area measure of deprivation using 2001 and 2011 census data from Scotland^☆^

**DOI:** 10.1016/j.healthplace.2016.03.006

**Published:** 2016-05

**Authors:** Mirjam Allik, Denise Brown, Ruth Dundas, Alastair H. Leyland

**Affiliations:** a Urban Big Data Centre, University of Glasgow, 7 Lilybank Gardens, Glasgow G12 8RZ, Scotland; b MRC/CSO Social and Public Health Sciences Unit, University of Glasgow, 200 Renfield Street, Glasgow G2 3QB, Scotland

**Keywords:** Deprivation, Health inequalities, Mortality, Carstairs deprivation score, Age, Scotland

## Abstract

Material deprivation contributes to inequalities in health; areas of high deprivation have higher rates of ill-health. How deprivation is measured has a great impact on its explanatory power with respect to health. We compare previous deprivation measures used in Scotland and proposes a new deprivation measure using the 2001 and 2011 Scottish census data. We calculate the relative index of inequality (RII) for self-reported health and mortality. While across all age groups different deprivation measures provide similar results, the assessment of health inequalities among those aged 20–29 differs markedly according to the deprivation measure. In 2011 the RII for long-term health problem for men aged 20–24 was only 0.71 (95% CI 0.60–0.83) using the Carstairs score, but 1.10 (0.99–1.21) for the new score and 1.13 (1.03–1.24) for the income domain of Scottish Index of Multiple Deprivation (SIMD). The RII for mortality in that age group was 1.25 (0.89–1.58) for the Carstairs score, 1.69 (1.35–2.02) for the new measure and 1.76 (1.43–2.08) for SIMD. The results suggest that researchers and policy makers should consider the suitability of deprivation measures for different social groups.

## Highlights

• Three measures of deprivation are associated with health similarly across all ages.
• The Carstairs score predicts lower health inequalities for people aged 20–29.
• The different result for the youth is driven by car ownership and overcrowding.

## 1 Introduction

Area-level measures of material deprivation are important for understanding and describing health inequalities (Adhikari, 2006, Barnett et al., 2001, Krieger et al., 2003) and are sometimes used by governments in an attempt to focus funding on the most disadvantaged communities (Welsh Government, 2011). These measures are also used when individual level variables of socioeconomic position are not available or when researchers wish to show that the deprivation of a place has an independent effect on a person's health beyond that of the individual socioeconomic circumstances (Ellaway et al., 2012, Meijer et al., 2012, Pickett and Pearl, 2001, Diez-Roux et al., 1997, Diez-Roux, 2004). The effectiveness of a deprivation measure in achieving these goals depends on how well it reflects the construct one wishes to measure (Diez-Roux, 1998) and on its validity for any particular social group or geographic area (Braveman et al., 2005, Martin et al., 2000).

For a long time the Carstairs deprivation score was one of the most frequently used measures of deprivation in Scotland and a basis for similar scores for the rest of UK. It is the only score in Scotland that has been provided over a 30-year span (1981–2011) (Brown et al., 2014). The availability of the Carstairs score for such a long time-span is a great strength, but has also made it vulnerable to social change. This has caused some debate about whether the index is still able to measure deprivation as well as it did in the past (Tunstall et al., 2011, Reid, 2009, Hanlon et al., 2005).

While new measures, such as the Scottish Index of Multiple Deprivation (SIMD), have been developed, there is still need for census-based measures of deprivation such as the Carstairs score. Measures using census data allow for a better comparison across countries and over time. Indices using policy take-up rates are less useful for comparative work as policies differ across countries and are changed by government. For example, welfare reform and the subsequent changes to benefits systems will mean that the income and employment domains of the SIMD have to be substantially revised. While census questions also change, they remain relatively constant over time and across different areas both within the UK and internationally, such that a reasonable comparison across decades or countries is possible. For this reason larger comparative studies prefer census based measures of deprivation (Exeter, 2011, Norman et al., 2011, Marí-Dell'Olmo et al., 2015). Constancy of questions also means that older census results can be used to derive new deprivation measures without loss of continuity across time. In addition, census data are provided for multiple different geographies (e.g. output areas, datazones and postcode sectors in Scotland) and as such census based measures can be easily replicated for different levels of analysis.

In this article we propose an alternative measure of deprivation using data from the 2001 and 2011 Scottish censuses. We show that in some circumstances the Carstairs score is unable to distinguish between deprived and less deprived areas and to detect potential health inequalities among young adults. In developing the new measure we draw on previous research on small-area deprivation measures and focus on wider applicability across different social groups and geographic areas. Finally, we compare the association of the new measure, the Carstairs score and the SIMD income domain to self-reported measures of health and mortality.

## 2 Measures of material deprivation

In the UK area level measures of deprivation have been used for decades to explain variation in health outcomes. The earlier measures included the Carstairs score (Carstairs and Morris, 1991), the Townsend (Townsend et al., 1988) and Jarman indices (Jarman, 1983). Since 2000 the Scottish, Welsh and Northern Irish governments have developed official measures of deprivation using administrative data – the SIMD (The Scottish Government, 2012), the Welsh Index of Multiple Deprivation (WIMD) (Welsh Government, 2011) and the Northern Ireland Multiple Deprivation Measure (NIMDM) (Northern Ireland Statistics and Research Agency, 2010) respectively. The Social Disadvantage Research Centre at the University of Oxford has produced the Indices of Deprivation (ID) for England since 2000 (McLennan et al., 2011). Similar measures of deprivation have also been developed in Australia (Pink, 2013), USA (Butler et al., 2013), New Zealand (Salmond and Crampton, 2012), Canada (Pampalon et al., 2009) and other countries (Havard et al., 2008, Maier et al., 2012, Panczak et al., 2012).

The national indices vary in some details depending on definitions, and how the various items are weighted and combined into a single index. In some countries the index may also include social deprivation (e.g. in Australia and Canada), which can refer to family relations or household type (e.g. single parent families, lone person households) and demographic or ethnic characteristics of the community (e.g. level of English). While social characteristics can have a strong association with health, they are clearly distinct from material deprivation – socially deprived people or areas are not necessarily materially deprived and vice versa. We have focused on material deprivation as it may be more amenable to policy interventions compared to social deprivation.

Despite some differences in these measures, there is widespread consensus on the main areas of material deprivation that should be included. These common items are (un-)employment, material wealth such as car ownership or income, indicators of socioeconomic position, particularly education and occupation, and housing conditions, such as overcrowding, home ownership or renting from a public authority. The Carstairs score combines four of these variables – no car ownership, male unemployment, overcrowding and low social class (Carstairs and Morris, 1991, McLoone, 1994, McLoone, 2004, Brown et al., 2014).

There are strong theoretical reasons for including each of these common indicators in a measure of material deprivation (see for example Galobardes et al., 2006a, Galobardes et al., 2006b). Material resources or wealth affects access to a number of factors that directly influence health (food, housing, various services, etc.). Wealth can be measured by income, but this is not an option when the census does not ask this question, as in Scotland. In such cases researchers often use proxies, like car ownership (Carstairs and Morris, 1991, Townsend et al., 1988). While frequently used as an indicator of wealth its validity is criticized by researchers interested in rural areas, where owning a car is not an indicator of material wealth, but rather a necessity (Farmer et al., 2001, Martin et al., 2000), the cost of which may further impoverish poor families (Barnett et al., 2001). Car ownership is not necessarily a better measure in urban areas, where not having a car might be a life style choice, even when affordable (Johnson et al., 2010). Ultimately, the ownership of any durable goods is a problematic indicator of wealth as it depends on preferences (McKay, 2004).

Occupation can affect health directly through the work environment (e.g. toxins) or the physical demands in places on employees, and indirectly, e.g. through material resources or social connections. Occupation based measures are easily available and frequently used, but vary over time due to changes in the occupational structures. The “low social class” used in the Carstairs score was based on Registrar General's social class classification (Carstairs and Morris, 1991). Due to the increases in service jobs and in women's labour force participation this classification became conceptually outmoded and was replaced in 2000 with the National Statistics Socio-economic Classification (NS-SeC) which is based on employment relations and conditions (Rose and Pevalin, 2005). For the Carstairs scores this means that retaining the old definition of low social class has become technically more difficult as well as theoretically less suitable. Another drawback of occupational measures is that they are sometimes not assigned to those currently not employed (as was the case in Scotland in 2001). Excluding the non-employed (e.g. temporarily unemployed, retired, those looking after a family, sick or disabled) from a deprivation measure may bias the results since mortality rates and mortality differentials among the inactive can be different from the economically active (Martikainen and Valkonen, 1999). In 2011 rules were used to estimate a category of NS-SeC for any person who did not have current occupation details and in most cases these people were classified according to their last main job (Scotland's Census, 2015b).

Exclusion from the labour market is also related to health through a variety of mechanisms, such as lack of resources, social isolation, stress and loss of self-esteem. A frequently used measure of exclusion from the labour market is unemployment. As a measure unemployment has some limitations, particularly in weak labour market conditions where it may undercount the true extent of labour market exclusion due to “hidden unemployment” – the diversion of people with health problems from recorded unemployment to recorded sickness (Beatty et al., 2000). Regardless the measure is still frequently used due to its association with health and its availability. While most deprivation measures use both male and female unemployment (e.g. SIMD or in Australia and New Zealand) the Carstairs score uses only male unemployment.

The effect of educational qualifications on material deprivation is nuanced. Education is associated with deprivation as the skills people obtain in school affect their employment, occupation and income. In addition education is unique in that it can capture the opportunities and constraints of childhood socio-economic conditions, which can have a strong impact on health outcomes later in life (Davey Smith et al., 1998, Galobardes et al., 2007, Lynch and Kaplan, 2003). Thus, not only does education correlate with other aspects of material deprivation, but it can differentiate between childhood opportunities for those who have the same deprivation level with respect to other indicators (e.g. employment) in adulthood. This is very important as current health is very much a product of life-long circumstances. Of the different areas of material deprivation widely included in measures, educational qualifications is the only one absent from the Carstairs score.

The lack of adequate housing also reflects material deprivation. Housing can be inadequate due to overcrowding (as in Carstairs score, SIMD and WIMD) or a lack of certain amenities such as central heating (as in WIMD and SIMD). Some people may be unable to afford housing at all and thus indicators of housing affordability and assistance (as in English ID) or housing tenure, specifically social renting (as in Australia) can also be used. Social renting was considered as an indicator of deprivation by Morris and Carstairs (1991), but since the proportion of public sector housing was at the time very high, it was excluded due to insufficient variation. Instead, overcrowding was used in creating Carstairs score, as well as in the Jarman and Townsend indices. Today overcrowding is a marginal issue, affecting only 3% of the Scottish population (Brown et al., 2014).

All the above are reasonable and generally good indicators of material deprivation, but their effect on inequalities in health can vary substantially among specific social groups. For example, the effect of income on health differences can depend on age (Mather et al., 2014, Robert and House, 1996) and the effect of education on health can depend on race/ethnicity (Braveman et al., 2005). Area deprivation also associates with high risk differences in adverse birth outcomes among white women, while the relationship between deprivation and adverse birth outcomes among black women is not as consistent (Messer et al., 2006). The health differences predicted by various deprivation measures can diverge enough to reach different conclusions about the magnitude, significance and even the sign of the effect (Braveman et al., 2005). But measures of deprivation should be robust across age, gender, ethnicity, geographic location and other factors. Therefore, it is important to consider the relationship of each of the different indicators on health by age or other social groups. To date, little is known about how the different deprivation measures compare with respect to age in Scotland.

While striving for better measures is important, deprivation is unlikely to be the only explanation for health inequalities. Walsh et al. (2010) find that even after taking account of deprivation, premature deaths in Glasgow are still higher than in Liverpool or Manchester. The authors conclude that even though deprivation has a strong impact on health, it is part of a complex picture. A variety of other hypothesis from genetic differences to climate have been posed to explain health inequalities, with some of the more plausible explanations focusing on a synthesis of negative health behaviours linked to lower social capital, de-industrialization and neoliberal policies (McCartney et al., 2012, Walsh et al., 2015).

## 3 Data and methods

### 3.1 The Carstairs deprivation score

The descriptions of the census variables used to calculate the Carstairs score (male unemployment, low social class, overcrowding and no car ownership) are shown in Table 1. To ensure that all components have an equal influence on the final score, each variable is standardised to have a population-weighted mean of zero and a variance of one (z-score method). The Carstairs score for each area is the sum of the standardised values (z-scores) of the components. The distributions and the population weighted means and standard deviations for the variables in 2011 are provided in Allik et al. (submitted for publication). The definition of the four variables has been kept as similar as possible across years. The scores have traditionally been calculated for postcode sectors but in 2011 they were also made available for datazones and output areas. We use the 2011 datazone level data published by Brown et al. (2014) and for 2001 we calculated the score for datazones using the same methodology.

### 3.2 The new deprivation measure

To create the new measure we also use datazone level data from the 2001 and 2011 Scottish censuses. In 2001 Scotland was divided into 6505 datazones with an average household population of 765 (sd 139) and in 2011 into 6500 datazones with an average household population of 799 (sd 261) people. All the new deprivation measure variables are calculated for the population living in private households. We tested a number of alternative specifications (such as different age limits) and chose those with higher correlations with health and steeper gradients for mortality. Correlations between some of the alternative specifications and the health variables are shown in the Supplement (Table S1). The descriptions of the census variables used to calculate the new measure are shown in Table 1.

To create the new measure we first suggest replacing male unemployment in the Carstairs score with overall unemployment. This is an improvement over the current measure as it takes account of female labour force participation. Unemployment is measured as the percentage of all economically active people aged 16–74 that are unemployed and seeking work. In 2011 the population weighted average unemployment rate across datazones was 8.3% (population weighted sd 5.1). The population weighted means, standard deviations and the distributions of variables and deprivation measures in 2011 are presented in Allik et al. (submitted for publication).

Secondly, low social class is replaced with an indicator of the position in the labour market based on the NS-SeC. The NS-SeC distinguishes three forms of employment relations, where the category “labour contract” captures those occupations where the employees are closely monitored and exchange discrete amounts of work for a wage (analytic classes 5–7). Typically this group includes the “working class” occupations, routine and semi-routine occupations (classes 6–7), but in weaker form also encompasses lower supervisory and technical occupations (class 5) (Rose and Pevalin, 2005). We propose using the percent of people in households where the household reference person (HRP) falls into analytic classes 6-7 to capture the aspects of deprivation related to labour market position. About 30% of Scottish people live in such households (sd 15). We excluded the analytic class 5 as this reduced the explanatory power of the variable on health and mortality (see Table S1).

We also recommend introducing tenure (percentage in socially rented accommodation) and educational qualifications (percentage with no qualifications) as new components. Over the last 30 years policy changes, such as the right to buy for public authority tenants, the decline in new local authority housing, and an increase of private sector building have dramatically changed housing tenure in Scotland. In 1984 approximately half of the Scottish population rented from a local authority or a housing association, by early 1990s this had reduced to about 40% and by 2000 to 30% (The Scottish Government, 2016). According to the 2011 census, about 20% of people live in social rented accommodations. Most datazones have no or few people in social rented accommodation, but in a small number of areas this percentage is very high. Priority for social housing is given to those threatened with homelessness, living in overcrowded households or in otherwise unsuitable accommodation, but medical, social or social work needs may also be taken into account. Given these considerations, it is likely that the more deprived people will have priority in receiving social accommodation. However, if policies around housing change, tenure, just like overcrowding, might become ineffective in capturing deprivation.

The second new component, educational qualifications, is measured as the percentage of people aged 16–74 with no school level educational qualifications. As education levels are strongly affected by age and gender we standardised the percentages by age and gender using the Scottish population at the census year. About a quarter of Scottish people aged 16–74 had no educational qualifications in 2011 (sd 11).

Between the two censuses changes to the classification of NS-SeC, economic activity and housing tenure were small and did not substantially affect the particular categories used here. Thus the comparison of these indicators between 2001 and 2011 should be fairly reliable. For more detailed information on the comparability of variables across censuses see metadata at Scotland's Census (2015a). There were more significant differences in the question on qualifications between the two censuses. In 2011 the census included categories “No qualifications” and “Other”, as opposed to the 2001 “None of these” category. Most people with no qualifications would have been expected to choose “None of these” in 2001 and “No qualifications” in 2011. However, those with foreign qualifications not listed among possible answers may have chosen “None of these”, in 2001 and “Other” in 2011. This could significantly affect the results for areas with high immigrant population. We use the percentage that chose “None of these” as an indicator of no qualifications for 2001 and the percent who chose “No qualifications” for 2011. For this reason the measures may not be directly comparable across the two years.

We have used the same methodology to calculate the new measure as is used for the Carstairs score, i.e. the measure is equal to the sum of equally weighted z-scores of the four components (see Brown et al. (2014) for details).

### 3.3 Health measures

To measure health at the area level we use 2001 and 2011 Scottish census data on self-reported health and death records collected by National Records of Scotland. We look at two indicators of self-reported health: the percentage of people who rate their general health as bad and the percentage who state they have a long-term illness. Mortality rates are calculated for all cause mortality between 2000 and 2002 and 2010–2012. We use census population counts as the denominator when calculating mortality rates. Both the health measures and the deaths are age-standardised using the 2013 European Standard Population (Eurostat, 2013).

The census questions and the variables used to measure general ill health and long-term illness are shown in Table 2. The questions vary over time, which may have also caused some difference in the overall levels of self-reported health. In 2001 the standardised average percent for people who rated their health as bad was 11.1% (sd 5.0) and for people with long-term illness 22.3% (sd 6.6). In 2011 these percentages were 6.1 (sd 3.5) and 10.2 (sd 4.5) respectively. The lower percentage of people with long-term illness in 2011 is most likely a consequence of the different answer choices presented in the census (see Table 2). For 2011 the percentage reflects those who felt their activities were limited a lot, but in 2001 it includes all those who felt their activities were limited, without a reference to any degree of limitation. For general health the question in 2001 refers to the last 12 months (unlike the 2011 question), which may have led people to consider their health over time, and possibly remember more instances of ill health, rather than give an assessment of the current state. For 2011 we also used the percentages of people who stated their health to be good or very good and those with no long-term illness for sensitivity analysis (selected results in Supplement Table S1). The average number of deaths per 100 people in a datazone was only 1.3 (sd 0.6) per year in 2001 and 1.1 per year in 2011 (sd 0.7).

### 3.4 Statistical methods

For 2001 the health data were provided for 15-year age groups and we only calculated the overall percentage of individuals in bad health and with long-term illness for each datazone. For 2011 we also analysed health outcomes by 5-year age groups. Because deaths are rare events we were unable to calculate mortality rates reliably for individual datazones. Instead we calculated mortality rates for deprivation deciles. Deciles are population-weighted and each includes 10% of the household population. Mortality rates were calculated for all ages and 5-year age groups.

To analyse the effect of deprivation on mortality and ill-health we mostly use the relative index of inequality (RII). The RII is obtained through the slope index of inequality (SII), which attempts to estimate the absolute health differences between the most and least deprived individuals in the society. The SII is simply the slope coefficient of an ordinary least squares regression, where the dependent variable is a health outcome and the independent variable a deprivation measure. The larger the slope coefficient the greater the impact of deprivation on health. As an absolute measure the SII is sensitive to differences in the mean level of population health and thus it is often divided by the mean level of population health, giving the RII (see Regidor (2004) for details). The higher values of RII indicated higher health inequalities with more deprived areas suffering poorer health than the least deprived areas. The 95% confidence intervals of the RII provide us with an estimate of uncertainty about health inequalities and were calculated using a multinomial simulation method described by Lumme et al. (2015). When the confidence intervals include zero the health differences between the least and most deprived areas are not statistically significant, i.e. we do not have enough certainty to state that health inequalities exist.

We have compared the effect of the new measure and its components to that of the Carstairs score, its components and also the income domain rank of the SIMD. We used the income domain rather than the full SIMD as the latter includes a health domain which is calculated using mortality rates. The income domain is highly correlated with the full SIMD ranking. For the 2001 analysis we have used SIMD 2004 where most income domain indicators are from 2002 and some from 2001. For 2011 we have used SIMD 2012 where most income domain indicators are from 2011 and one from 2010.

We used the Scottish Government 2-fold urban rural classification to define rurality (The Scottish Government, 2015b). Settlements of 3000 or more people are classified as urban and settlements below this as rural. This classification identifies 1326 (20.4%) rural datazones with 19.6% of population in 2001 and 1175 (18.1%) datazones with 18.5% of population in 2011. We were unable to use the more detailed classifications as the number of different rural datazones becomes too small. All data on deprivation scores, self-reported health, urban-rural classification, and population breakdown by age and gender for 2001 and 2011 are available from Allik et al. (submitted for publication).

Since the true level of health inequalities is unknown we assess the measures based on their ability to distinguish between areas with different health outcomes. If one of the three measures or their constituent variables shows much lower health inequalities than the others it can indicate the ineffectiveness of this particular measure in picking up area-level deprivation and health inequalities.

## 4 Results: the effect of material deprivation on health and mortality

### 4.1 Self-reported health and mortality across all ages

**Fig. 1:**
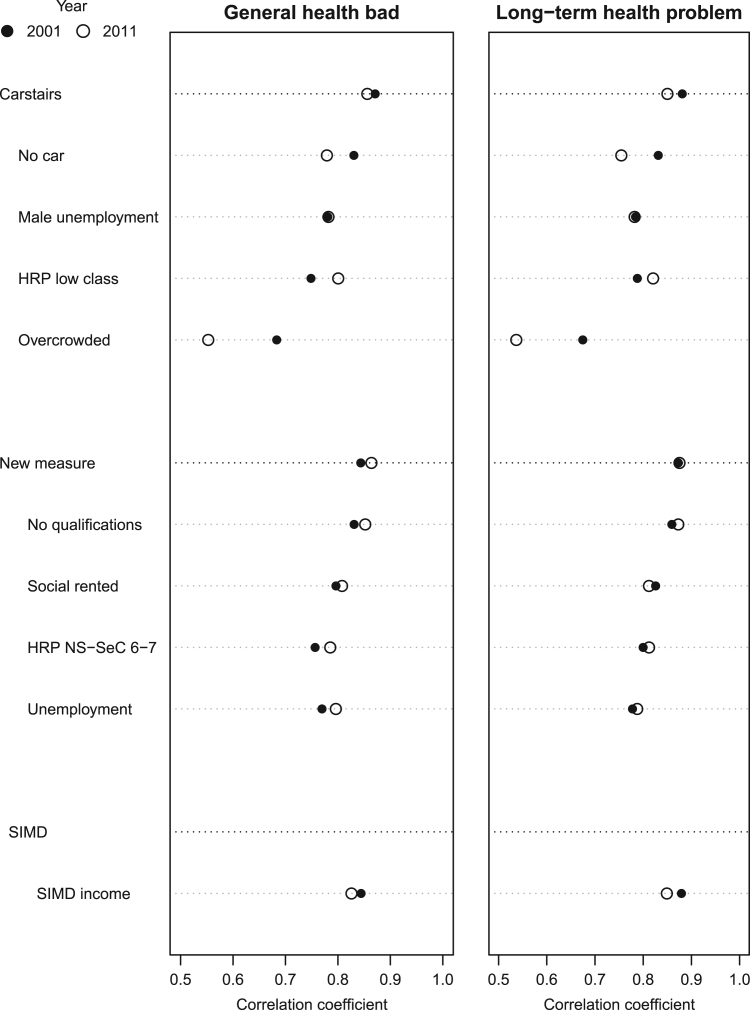
Correlation between deprivation and self-reported health, 2001 and 2011.

Fig. 1 shows the correlation coefficients between the indicators of material deprivation and self-reported health (men and women of all ages) for 2001 and 2011. The three deprivation measures are correlated with self-reported ill-health equally well, the coefficients for general bad health are between 0.83 and 0.87 and for long-term health problems between 0.85 and 0.88. However, there are considerable differences in how the individual indicators of the Carstairs score are associated with health, particularly, the coefficient for overcrowding, which has visibly decreased in size and was only about 0.55 in 2011. While some variation between the different indicators and over time could be expected, the correlation coefficients for overcrowding suggest that the variable has become less suited to capture material deprivation and explain ill-health.

Table 3 shows all cause mortality rates for men (all ages) by the three measures and the components of two of them. For 2001 the Carstairs score and the new measure give similar mortality rates and measures of inequality, RII 0.61 (95% CI 0.59–0.64) and 0.59 (95% CI 0.56–0.61) respectively. The income domain of the SIMD suggests greater inequalities, RII 0.69 (95%CI 0.67–0.72). For 2011 the three measures all suggest that mortality has decreased for both the least and most deprived deciles, but that the RII has increased to approximately 0.7 for all three measures. Looking at the individual components of the Carstairs score we see that inequalities in mortality are much lower for overcrowding, RII 0.5 (0.47–0.53) in 2011. Unlike the other indicators overcrowding does not indicate an increase in inequalities between 2001 and 2011.

The patterns shown in Table 3 are similar for women – all measures show that mortality has decreased for both the most and least deprived deciles, but inequalities have increased. In 2001 the RII for the Carstairs score was 0.38 (0.35–0.40) and for the new measure 0.37 (0.35–0.40), and 0.50 (0.48–0.53) for the SIMD. In 2011 the three measures give very similar results and the RII ranges between 0.55 and 0.59. Of the different variables inequalities are lowest by overcrowding (RII in 2011 0.44 95% CI 0.42–0.47).

Together these results show that across all ages the three measures give very similar results and are equally useful predictors of ill-health and mortality. However, the analysis also suggests that overcrowding is not a good predictor of ill-health or mortality. Table 3 shows that excluding overcrowding from the Carstairs score affects mortality rates only marginally and does not reduce the RII. Neither does this exclusion reduce the correlation coefficient between the Carstairs score and self-reported health (Supplement Table S1).

We also looked at the relationship between the deprivation variables and health and mortality in urban and rural areas separately (Supplement Tables S2 and S3). All indicators have weaker associations with self-reported health and lower gradients for mortality in rural areas. Of the three measures the new score has the most similar associations between health and deprivation in the urban and rural areas. In 2011 the correlation coefficients between long-term illness and the new score are 0.87 and 0.80 in urban and rural areas respectively. For the income domain of SIMD these are 0.85 and 0.78, and for the Carstairs score somewhat lower at 0.85 and 0.74. The results for all-cause male mortality are similar. The RII for urban and rural areas are 0.66 and 0.55 respectively using the new measure, 0.70 and 0.56 for the income domain of SIMD and 0.68 and 0.52 for the Carstairs score. However, the presented results for RII in rural areas should be taken with caution as in many cases very few datazones fall into deprived deciles. In 2011 only 6 (0.5%) rural datazones are in the most deprived Carstairs decile and using only car ownership none of the rural datazones are in the most deprived decile. Using the new measure we get 16 (1.4%) and with SIMD income domain 12 (1.0%) most deprived rural datazones.

### 4.2 Self-reported health and mortality by age groups

**Fig. 2:**
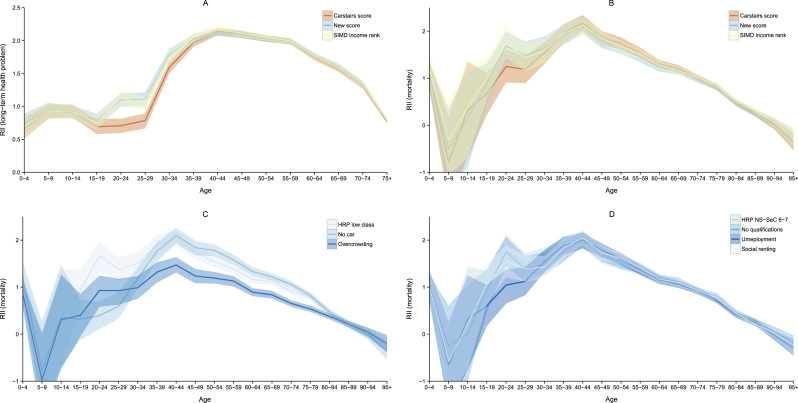
Relative index of inequality for three deprivation measures and their components, men 2011.

Fig. 2 panel A shows RII and the 95% confidence intervals for self-reported long-term health problems for men by 5-year age groups. For most ages the three measures give very similar results – the indices and the confidence intervals overlap. However, for ages 15–19 and 35–39 the Carstairs score is associated with somewhat lower inequalities and for ages 20–34 substantially lower inequalities in long-term health. The differences are greatest for ages 20–24, while the RII using the Carstairs score is 0.71 (95% CI 0.60-0.83), it is 1.10 (95% CI 0.99–1.21) for the new measure and 1.13 (95% CI 1.03–1.24) for the SIMD income domain. For women the RII at ages 20–24 is 0.43 (0.29–0.55) for the Carstairs score, but 0.84 (0.72–0.95) for the new measure and 0.86 (0.75–0.98) for the SIMD income domain. (See Tables S4–S6 in the Supplement for the RII for both men and women by age for self-reported bad health, long-term illness and mortality in 2011).

We see a similar result when plotting the RII for male mortality as in panel B of Fig. 2. The Carstairs score suggests lower inequalities for ages 15–19, and particularly for ages 20–29. The confidence intervals in this case are larger and overlap. This pattern remains largely the same for 2001 male and 2001 and 2011 female mortality.

We looked at the predicted RII of the different components of the Carstairs score to determine what drives the low health inequalities among the young adults. Panel C of Fig. 2 shows that no car ownership predicts the lowest inequalities for ages 20–29. Using car ownership the RII for men aged 20–24 is only 0.40 (95% CI 0.07–0.69), while low social class of the HRP produces a RII about four times as high, 1.67 (95% CI 1.31–1.99). Since the RII and CI for male unemployment roughly overlap with the indicator for low class they were excluded from the plot for clarity. This pattern is repeated for women – in 2011 the RII for women aged 20–24 was only 0.28 (−0.20 to 0.79) using car ownership, but 1.17 (0.68–1.64) using low social class of HRP. Thus we would have arrived at different conclusions about the extent and significance of inequalities in mortality. Panel C also shows that inequalities are lower by overcrowding, and consistently so, suggesting again that it is probably not a good marker of health inequalities. The same applies for mortality among women and for the year 2001.

Finally, panel D of Fig. 2 shows the RII and the confidence intervals for the different variables of the new deprivation measure. There is some variation in the predicted inequalities for ages 20–29. Unemployment suggests that inequalities in mortality are lower among those ages, but the difference is smaller and does not affect the overall measure very much. As a result the new measure suggests that inequalities are higher for ages 20–29 than indicated by the Carstairs score.

## 5 Discussion

Three different measures of material deprivation – the Carstairs score, the income domain of SIMD and the new measure – are associated with self-reported ill-health and mortality similarly at the population level. However, major differences between the three measures emerge when we analyse the effect of deprivation on health and mortality by age groups. The Carstairs score is less able to distinguish health inequalities among young adults aged 20–29. This is largely driven by including car ownership in the measure, but accentuated by overcrowding which under predicts inequalities for nearly all ages.

Questions about the ability of Carstairs score to measure deprivation have been raised (Reid, 2009, Tunstall et al., 2011, Hanlon et al., 2005), with the debate mostly focusing on the effectiveness of car ownership as an indicator of deprivation in rural areas (Johnson et al., 2010, Christie and Fone, 2003, Farmer et al., 2001, Martin et al., 2000). Our study adds to this debate by showing that car ownership may also underestimate ill health and health inequalities among youth, in addition to the urban-rural issues already raised.

Compared to the Carstairs score the new measure is theoretically more sound given the social (e.g. reduction in overcrowding) and labour market (e.g. increase in female labour force participation) changes since 1981. This more current theoretical perspective means that the new measure is better able to distinguish between areas of poor and good health compared to the Carstairs score. Compared to the SIMD the new measure is just as effective in capturing deprivation and health inequalities, but has the added benefit of being available for multiple decades in much the same way. Further advantages of the new measure over SIMD are that it consists of only 4 variables compared to 38 for the full SIMD, and the calculation procedure from raw data to score is much simpler for the new measure compared to SIMD (The Scottish Government, 2012). As a census based measure it is not affected by policy change (which the SIMD is) and is available for different geographies, particularly output areas. Of course, the new measure is not completely robust to potential future social change and is dependent on the continuation of the census and changes in the questionnaire.

From an international perspective the work here shows that more attention should be paid to the robustness of small-area deprivation measures with respect to different social groups and/or geographic location. Many of the often used variables (e.g. education) can be appropriate for different age groups, but others (car ownership, overcrowding) are likely to be ineffective in distinguishing between deprivation levels in other countries just as well. The small-area index of deprivation developed for France includes indicators of car ownership and overcrowding but does not assess any differential effects on health inequalities by age group (Havard et al., 2008). A study in the US has found that an area deprivation indicator based on overcrowding is not statistically significantly associated with physical and mental health scores and the number of chronic conditions while controlling for age (Eibner and Sturm, 2006).

Similarly to Pampalon et al. (2009) we also found that all deprivation variables produced lower inequalities in mortality in rural areas compared to urban areas, suggesting that researchers should also consider this aspect in developing measures for countries with substantial rural populations. This issue has been highlighted in the UK (Farmer et al., 2001, Martin et al., 2000) and in developing the SIMD (The Scottish Government, 2015a), but appropriate solutions are still being developed and may vary between countries. The current work has not tested the robustness of the different variables with respect to race, ethnicity and many other social characteristics, but this should clearly be a focus in the future, especially in diverse countries (Braveman et al., 2005, Messer et al., 2006, Tunstall et al., 2011).

For policy makers these results highlight the importance of using new and varied deprivation measures to understand health inequalities. Relying on one measure, even if as established as the Carstairs score, may not be able provide a full picture of a complex problem. But the work also shows that older census results can be used to derive new measures and thus the continuity across time does not have to be lost with the development of new measures.

## Figures and Tables

**Table 1:** Census variables used to create Carstairs score and the new score.

Carstairs score			New measure	
Variable	Description		Variable	Description
Male unemployment	Economically active males aged 16–74 seeking or waiting to start work, as a proportion of all economically active males 16–74		Unemployment	Economically active persons aged 16–74 in households seeking or waiting to start work, as a proportion of all economically active people (16–74) in households
Low social class	Persons living in households with the HRP (aged 16–74) in social class IV or V, as a proportion of all people in households with an economically active HRP (16–74)		NS-SeC 6–7	Persons living in households with the HRP (aged 16–74) in NS-SeC 6–7, as a proportion of all people in households with an economically active HRP (16–74)
Overcrowding	Persons living in households at a density of more than one person per room, as a proportion of all people in households		Social renting	Persons in households renting from social landlord, as a proportion of all people in households
No car ownership	Persons living in households with no car, as a proportion of all people in private households			
			No qualifications	Persons with no school level educational qualifications aged 16–74, as a proportion of all people (16–74), age and gender standardised


**Table 2:** Census questions on health used to measure bad health and long-term illness.

Year	Question and answer choices	Variable used
*General health*		
2011	How is your health in general?	Percent “Bad”
	Very good, Good, Fair, Bad, Very bad	and “Very bad”
2001	Over the last 12 months would you say your health has on the whole been:	Percent “Not good”
	Good? Fairly good? Not good?	

*Long-term health*		
2011	Are your day-to-day activities limited because of a health problem or disability which has lasted, or is expected to last, at least 12 months? Include problems related to old age.	Percent “Yes, limited a lot”
	Yes, limited a lot. Yes, limited a little. No	
2001	Do you have any long-term illness, health problem or disability which limits your daily activities or the work you can do? Include problems which are due to old age. Yes, No	Percent “Yes”

**Table 3:** All cause mortality for the least and most deprived deciles and RII by deprivation measure, men 2001 and 2011.

Variable	2001					2011			
	Least	Most	RII	95% CI		Least	Most	RII	95% CI
**New measure**	**1284**	**2301**	**0.59**	**(0.56–0.61)**		**1003**	**1958**	**0.69**	**(0.67–0.72)**
No qualifications	1282	2284	0.58	(0.55–0.60)		1038	1953	0.67	(0.64–0.70)
Social rented	1335	2265	0.57	(0.54–0.59)		1037	1947	0.66	(0.63–0.68)
HRP NS-SeC 6–7	1316	2168	0.52	(0.50–0.55)		1019	1893	0.64	(0.62–0.67)
Unemployment	1380	2272	0.54	(0.51–0.56)		1101	1902	0.63	(0.60–0.65)
									
**Carstairs score**	**1334**	**2353**	**0.61**	**(0.59–0.64)**		**1029**	**1977**	**0.70**	**(0.67–0.73)**
No car	1373	2334	0.60	(0.57–0.63)		1047	1942	0.69	(0.67–0.72)
HRP low class	1308	2217	0.53	(0.50–0.55)		1002	1916	0.65	(0.62–0.68)
Male unemployment	1377	2310	0.54	(0.51–0.57)		1119	1944	0.61	(0.58–0.64)
Overcrowding	1386	2226	0.49	(0.47–0.52)		1129	1815	0.50	(0.47–0.53)
Carstair excl. overcrowding	1291	2349	0.61	(0.58–0.63)		1033	1969	0.70	(0.67–0.72)
									
**SIMD income rank**	**1211**	**2396**	**0.69**	**(0.67–0.72)**		**995**	**1985**	**0.73**	**(0.70–0.75)**

## Appendix A Supplementary data

Application 1
