# Supplementary material for: Developing a new small-area measure of deprivation using 2001 and 2011 census data from Scotland
Source: Health Place. 2016 May;39:122–30. doi: 10.1016/j.healthplace.2016.03.006 (PMC4889779; doi:10.1016/j.healthplace.2016.03.006)
Supplement: Application 1 [file mmc1.pdf]

Supplementary tables and figures

Table S.1: Correlation between deprivation variables and self reported health, 2001 and 2011

Variable	2001		2011			
	Bad health	Long-term illness	Bad health	Good health	Long-term illness	No long-term illness
**New score**	**0.84**	**0.87**	**0.86**	**-0.91**	**0.88**	**-0.90**
Social rented	0.80	0.83	0.81	-0.85	0.81	-0.84
No qualifications (standardised, 16-74)	0.83	0.86	0.85	-0.90	0.87	-0.88
No qualifications (16-64)	0.76	0.80				
No qualifications (16-74)	0.74	0.79	0.76	-0.81	0.79	-0.80
HRP No qualifications	0.75	0.80				
Percent unemployed	0.77	0.78	0.80	-0.83	0.79	-0.81
Percent employed (25-54)	-0.86	-0.86				
Percent employed (25-49)	-0.84	-0.84				
HRP unemployed	0.74	0.74				
HRP NS-SeC 6-7	0.76	0.80	0.79	-0.85	0.81	-0.85
HRP NS-SeC 5-7	0.71	0.76				
Percent NS-SeC 6-7	0.71	0.76				
Percent NS-SeC 5-7	0.68	0.74				
**Carstairs score**	**0.87**	**0.88**	**0.86**	**-0.90**	**0.85**	**-0.88**
Percent unemployed men	0.78	0.78	0.78	-0.81	0.78	-0.80
Percent with no car	0.83	0.83	0.78	-0.80	0.75	-0.78
HRP low social class	0.75	0.79	0.80	-0.86	0.82	-0.85
Percent overcrowded	0.68	0.67	0.55	-0.57	0.54	-0.55
Carstairs excl. overcrowding	0.86	0.88	0.87	-0.91	0.86	-0.89
**SIMD income rank**	**0.84**	**0.88**	**0.83**	**-0.88**	**0.85**	**-0.88**

See Table 2 for definitions and census questions of bad health and long-term illness.

Table S.2: Correlation between deprivation variables and self reported health for urban and rural areas, 2001 and 2011

Variable	2001				2011			
	Bad health		Long-term ill		Bad health		Long-term ill	
	Urban	Rural	Urban	Rural	Urban	Rural	Urban	Rural
New score	0.84	0.77	0.88	0.79	0.86	0.79	0.87	0.80
Social rented	0.79	0.71	0.83	0.72	0.81	0.69	0.81	0.69
No qualifications	0.84	0.75	0.87	0.76	0.85	0.78	0.87	0.80
Unemployed	0.78	0.61	0.78	0.64	0.79	0.71	0.78	0.69
HRP NS-SeC 6-7	0.76	0.67	0.80	0.70	0.78	0.70	0.81	0.73
Carstairs score	0.87	0.75	0.88	0.77	0.85	0.73	0.85	0.74
Unemployed men	0.78	0.60	0.79	0.63	0.77	0.67	0.78	0.66
No car	0.82	0.73	0.83	0.75	0.76	0.71	0.74	0.72
HRP low class	0.76	0.62	0.80	0.66	0.80	0.66	0.82	0.68
Overcrowded	0.68	0.58	0.67	0.59	0.54	0.40	0.52	0.41
SIMD income rank	0.85	0.73	0.89	0.76	0.82	0.76	0.85	0.78

Table S.3: Relative index of Inequality for urban and rural areas, male mortality 2001 and 2011

Variable	2001				2011			
	Urban		Rural		Urban		Rural	
	RII	95% CI	RII	95% CI	RII	95% CI	RII	95% CI
New measure	0.58	(0.55-0.60)	0.42	(0.35-0.51)	0.66	(0.63-0.69)	0.55	(0.46-0.66)
No qualification	0.57	(0.55-0.60)	0.41	(0.33-0.48)	0.65	(0.62-0.67)	0.52	(0.43-0.62)
Social rented	0.56	(0.54-0.59)	0.37	(0.29-0.46)	0.64	(0.61-0.66)	0.43	(0.32-0.50)
HRP NS-SeC 6-7	0.52	(0.49-0.54)	0.35	(0.27-0.44)	0.62	(0.59-0.65)	0.50	(0.40-0.59)
Unemployment	0.55	(0.52-0.58)	0.24	(0.17-0.31)	0.62	(0.59-0.65)	0.39	(0.30-0.47)
Carstairs score	0.61	(0.58-0.64)	0.45	(0.36-0.54)	0.68	(0.65-0.71)	0.52	(0.42-0.63)
No car	0.63	(0.59-0.66)	0.51	(0.41-0.60)	0.71	(0.68-0.74)	0.47	(0.36-0.58)
HRP low class	0.53	(0.50-0.56)	0.30	(0.22-0.38)	0.63	(0.60-0.66)	0.43	(0.33-0.53)
Male unemployment	0.56	(0.53-0.58)	0.27	(0.19-0.34)	0.61	(0.58-0.64)	0.34	(0.26-0.42)
Overcrowding	0.48	(0.45-0.51)	0.41	(0.33-0.48)	0.48	(0.45-0.51)	0.33	(0.25-0.41)
SIMD income rank	0.69	(0.66-0.71)	0.55	(0.45-0.63)	0.7	(0.67-0.73)	0.56	(0.46-0.66)

Table S.4: RII for self-rated bad and very bad health, 2011

Age	Men						Women					
	Carstairs		New score		SIMD inc. rank		Carstairs		New score		SIMD inc. rank	
	RII	95% CI	RII	95% CI	RII	95% CI	RII	95% CI	RII	95% CI	RII	95% CI
0-4	1.13	(0.87-1.37)	1.07	(0.82-1.31)	1.09	(0.84-1.33)	1.14	(0.86-1.40)	1.10	(0.78-1.39)	1.14	(0.85-1.43)
5-9	1.49	(1.23-1.74)	1.43	(1.19-1.70)	1.45	(1.20-1.70)	1.53	(1.21-1.81)	1.40	(1.09-1.71)	1.46	(1.16-1.75)
10-14	1.33	(1.10-1.55)	1.32	(1.10-1.57)	1.33	(1.10-1.56)	1.45	(1.19-1.70)	1.35	(1.11-1.59)	1.42	(1.17-1.67)
15-19	1.29	(1.09-1.49)	1.25	(1.05-1.44)	1.22	(1.03-1.42)	0.99	(0.80-1.19)	0.98	(0.77-1.16)	1.04	(0.87-1.25)
20-24	1.25	(1.09-1.41)	1.37	(1.21-1.53)	1.44	(1.28-1.59)	0.82	(0.66-0.98)	1.09	(0.94-1.26)	1.13	(0.99-1.27)
25-29	1.38	(1.23-1.51)	1.58	(1.43-1.71)	1.56	(1.43-1.70)	1.13	(1.00-1.25)	1.34	(1.21-1.47)	1.29	(1.16-1.41)
30-34	1.77	(1.67-1.88)	1.89	(1.78-2.00)	1.86	(1.75-1.97)	1.60	(1.50-1.70)	1.77	(1.67-1.87)	1.72	(1.62-1.83)
35-39	2.20	(2.12-2.28)	2.23	(2.15-2.31)	2.23	(2.15-2.30)	1.84	(1.76-1.92)	1.89	(1.81-1.97)	1.87	(1.79-1.95)
40-44	2.33	(2.27-2.40)	2.30	(2.23-2.36)	2.34	(2.27-2.40)	1.99	(1.92-2.05)	2.00	(1.94-2.06)	1.99	(1.93-2.06)
45-49	2.30	(2.25-2.37)	2.27	(2.22-2.33)	2.30	(2.25-2.36)	2.00	(1.95-2.05)	2.00	(1.94-2.05)	2.00	(1.95-2.05)
50-54	2.17	(2.11-2.22)	2.16	(2.11-2.22)	2.18	(2.13-2.24)	2.01	(1.96-2.05)	2.00	(1.95-2.05)	2.00	(1.95-2.05)
55-59	2.15	(2.10-2.20)	2.14	(2.09-2.19)	2.15	(2.10-2.20)	1.98	(1.93-2.03)	1.99	(1.94-2.04)	1.98	(1.93-2.03)
60-64	1.93	(1.88-1.98)	1.96	(1.91-2.00)	1.95	(1.90-1.99)	1.92	(1.87-1.98)	1.93	(1.89-1.99)	1.93	(1.88-1.98)
65-69	1.79	(1.74-1.85)	1.80	(1.75-1.86)	1.80	(1.75-1.86)	1.75	(1.69-1.81)	1.77	(1.72-1.83)	1.77	(1.71-1.83)
70-74	1.55	(1.49-1.62)	1.54	(1.48-1.61)	1.57	(1.51-1.64)	1.55	(1.49-1.60)	1.55	(1.50-1.60)	1.57	(1.52-1.63)
75+	1.12	(1.07-1.16)	1.10	(1.05-1.14)	1.12	(1.08-1.17)	1.07	(1.03-1.11)	1.06	(1.02-1.09)	1.09	(1.06-1.13)
All ages	1.78	(1.76-1.8)	1.78	(1.76-1.8)	1.79	(1.77-1.81)	1.66	(1.64-1.67)	1.67	(1.65-1.69)	1.67	(1.65-1.69)

Table S.5: RII for self-rated long-term health problem, 2011

Age	Men						Women					
	Carstairs		New score		SIMD inc. rank		Carstairs		New score		SIMD inc. rank	
	RII	95% CI	RII	95% CI	RII	95% CI	RII	95% CI	RII	95% CI	RII	95% CI
0-4	0.67	(0.52-0.81)	0.74	(0.58-0.89)	0.69	(0.53-0.85)	0.75	(0.55-0.94)	0.75	(0.53-0.95)	0.73	(0.52-0.93)
5-9	0.93	(0.81-1.03)	0.95	(0.84-1.07)	0.93	(0.82-1.05)	0.92	(0.76-1.09)	0.93	(0.77-1.10)	0.93	(0.76-1.10)
10-14	0.93	(0.83-1.02)	0.91	(0.80-1.01)	0.92	(0.82-1.02)	1.01	(0.87-1.14)	0.99	(0.86-1.15)	1.01	(0.88-1.15)
15-19	0.69	(0.59-0.80)	0.79	(0.68-0.89)	0.75	(0.65-0.86)	0.66	(0.54-0.78)	0.73	(0.61-0.85)	0.77	(0.65-0.88)
20-24	0.71	(0.59-0.81)	1.10	(1.00-1.20)	1.13	(1.02-1.23)	0.43	(0.29-0.55)	0.84	(0.72-0.95)	0.86	(0.75-0.98)
25-29	0.78	(0.66-0.90)	1.11	(1.01-1.22)	1.04	(0.92-1.15)	0.79	(0.67-0.90)	1.07	(0.97-1.17)	1.02	(0.92-1.12)
30-34	1.60	(1.51-1.68)	1.79	(1.70-1.87)	1.75	(1.67-1.83)	1.43	(1.35-1.52)	1.62	(1.53-1.70)	1.56	(1.47-1.64)
35-39	1.97	(1.90-2.04)	2.03	(1.95-2.10)	2.05	(1.98-2.12)	1.71	(1.64-1.78)	1.78	(1.72-1.85)	1.77	(1.70-1.84)
40-44	2.14	(2.08-2.20)	2.13	(2.07-2.18)	2.17	(2.12-2.23)	1.80	(1.75-1.86)	1.83	(1.77-1.88)	1.83	(1.78-1.88)
45-49	2.10	(2.05-2.15)	2.09	(2.04-2.14)	2.11	(2.06-2.16)	1.81	(1.76-1.86)	1.82	(1.78-1.87)	1.83	(1.79-1.88)
50-54	2.03	(1.98-2.08)	2.03	(1.98-2.08)	2.05	(2.00-2.10)	1.85	(1.81-1.90)	1.87	(1.83-1.92)	1.86	(1.81-1.90)
55-59	1.99	(1.94-2.04)	1.99	(1.94-2.03)	1.99	(1.95-2.04)	1.75	(1.71-1.80)	1.78	(1.74-1.82)	1.75	(1.71-1.80)
60-64	1.75	(1.70-1.79)	1.78	(1.73-1.82)	1.76	(1.72-1.80)	1.64	(1.59-1.68)	1.67	(1.63-1.71)	1.66	(1.62-1.70)
65-69	1.57	(1.52-1.61)	1.61	(1.56-1.65)	1.59	(1.55-1.64)	1.51	(1.46-1.55)	1.53	(1.49-1.58)	1.53	(1.49-1.57)
70-74	1.32	(1.27-1.36)	1.34	(1.29-1.38)	1.33	(1.29-1.38)	1.28	(1.23-1.32)	1.30	(1.26-1.34)	1.31	(1.26-1.35)
75+	0.78	(0.75-0.81)	0.79	(0.75-0.82)	0.80	(0.77-0.83)	0.69	(0.67-0.72)	0.69	(0.67-0.71)	0.73	(0.70-0.75)
All ages	1.41	(1.39-1.42)	1.44	(1.42-1.45)	1.44	(1.42-1.45)	1.28	(1.27-1.30)	1.31	(1.30-1.33)	1.32	(1.31-1.33)

Table S.6: RII for all-cause mortality, 2011

Age	Men						Women					
	Carstairs		New score		SIMD inc. rank		Carstairs		New score		SIMD inc. rank	
	RII	95% CI	RII	95% CI	RII	95% CI	RII	95% CI	RII	95% CI	RII	95% CI
0-4	1.08	(0.81;1.35)	1.05	(0.75;1.35)	1.21	(0.91;1.5)	0.94	(0.60;1.29)	0.93	(0.58;1.29)	0.9	(0.54;1.29)
5-9	-0.78	(-1.67;0.06)	-0.50	(-1.36;0.27)	-0.32	(-1.16;0.57)	0.70	(-0.46;1.99)	0.95	(-0.03;2.01)	1.11	(-0.08;2.23)
10-14	0.33	(-0.67;1.32)	0.14	(-0.93;1.20)	0.38	(-0.63;1.28)	0.42	(-0.68;1.48)	0.64	(-0.43;1.75)	0.98	(-0.10;2.09)
15-19	0.67	(0.19;1.11)	0.90	(0.45;1.33)	1.00	(0.55;1.46)	0.67	(-0.02;1.26)	0.68	(0.01;1.26)	0.86	(0.25;1.48)
20-24	1.25	(0.89;1.58)	1.69	(1.35;2.02)	1.76	(1.43;2.08)	0.92	(0.40;1.47)	1.13	(0.61;1.63)	1.35	(0.81;1.80)
25-29	1.19	(0.88;1.46)	1.49	(1.19;1.77)	1.43	(1.12;1.71)	1.11	(0.69;1.53)	1.29	(0.88;1.71)	1.19	(0.76;1.65)
30-34	1.54	(1.29;1.78)	1.64	(1.42;1.88)	1.59	(1.32;1.82)	1.05	(0.68;1.45)	1.11	(0.74;1.49)	1.19	(0.79;1.55)
35-39	1.96	(1.76;2.15)	1.93	(1.71;2.13)	1.95	(1.74;2.18)	1.50	(1.22;1.79)	1.53	(1.22;1.84)	1.46	(1.15;1.75)
40-44	2.17	(2.01;2.33)	2.11	(1.94;2.28)	2.13	(1.98;2.29)	1.61	(1.40;1.82)	1.57	(1.35;1.79)	1.58	(1.38;1.79)
45-49	1.86	(1.71;2.00)	1.79	(1.65;1.94)	1.85	(1.71;2.00)	1.27	(1.08;1.46)	1.31	(1.12;1.49)	1.33	(1.15;1.52)
50-54	1.74	(1.60;1.85)	1.64	(1.51;1.78)	1.66	(1.54;1.78)	1.33	(1.17;1.48)	1.34	(1.20;1.50)	1.35	(1.19;1.51)
55-59	1.54	(1.43;1.64)	1.45	(1.34;1.55)	1.52	(1.41;1.63)	1.27	(1.13;1.40)	1.24	(1.11;1.38)	1.30	(1.16;1.44)
60-64	1.30	(1.21;1.39)	1.23	(1.15;1.31)	1.28	(1.19;1.37)	1.19	(1.08;1.29)	1.17	(1.05;1.27)	1.16	(1.05;1.27)
65-69	1.20	(1.12;1.27)	1.16	(1.08;1.24)	1.16	(1.08;1.24)	1.15	(1.06;1.25)	1.15	(1.05;1.24)	1.14	(1.04;1.24)
70-74	1.00	(0.93;1.08)	0.98	(0.91;1.05)	1.01	(0.95;1.09)	0.97	(0.88;1.04)	0.96	(0.88;1.03)	0.99	(0.92;1.07)
75-79	0.81	(0.74;0.87)	0.82	(0.76;0.88)	0.84	(0.78;0.91)	0.74	(0.68;0.80)	0.75	(0.69;0.82)	0.78	(0.72;0.85)
80-84	0.48	(0.43;0.54)	0.46	(0.41;0.53)	0.51	(0.45;0.57)	0.36	(0.31;0.42)	0.39	(0.34;0.45)	0.43	(0.37;0.49)
85-89	0.26	(0.19;0.33)	0.26	(0.19;0.33)	0.31	(0.25;0.39)	0.20	(0.14;0.26)	0.22	(0.17;0.28)	0.26	(0.20;0.31)
90-94	0.01	(-0.09;0.12)	0.05	(-0.05;0.14)	0.07	(-0.02;0.17)	-0.08	(-0.15;-0.02)	-0.06	(-0.13;0.00)	-0.03	(-0.10;0.03)
95+	-0.36	(-0.54;-0.18)	-0.25	(-0.42;-0.08)	-0.24	(-0.44;-0.05)	-0.08	(-0.18;0.01)	-0.04	(-0.13;0.06)	-0.02	(-0.12;0.08)
All ages	0.70	(0.67;0.72)	0.69	(0.66;0.72)	0.73	(0.70;0.75)	0.55	(0.53;0.58)	0.57	(0.55;0.60)	0.59	(0.57;0.62)
